# Emotional Granularity Increases With Intensive Ambulatory Assessment: Methodological and Individual Factors Influence How Much

**DOI:** 10.3389/fpsyg.2021.704125

**Published:** 2021-07-28

**Authors:** Katie Hoemann, Lisa Feldman Barrett, Karen S. Quigley

**Affiliations:** ^1^Department of Psychology, Katholieke Universiteit, Leuven, Belgium; ^2^Department of Psychology, Northeastern University, Boston, MA, United States; ^3^Department of Psychiatry, Harvard University, Boston, MA, United States; ^4^Athinoula A. Martinos Center for Biomedical Imaging, Massachusetts General Hospital, Charlestown, MA, United States; ^5^Bedford VA Medical Center, Bedford, MA, United States

**Keywords:** emotional granularity, emotion differentiation, experience sampling, ecological momentary assessment, daily diary, ambulatory assessment, intervention

## Abstract

Individuals differ in their ability to create instances of emotion that are precise and context-specific. This skill – referred to as *emotional granularity* or *emotion differentiation* – is associated with positive mental health outcomes. To date, however, little work has examined whether and how emotional granularity might be increased. Emotional granularity is typically measured using data from experience sampling studies, in which participants are prompted to report on their emotional experiences multiple times per day, across multiple days. This measurement approach allows researchers to examine patterns of responses over time using real-world events. Recent work suggests that experience sampling itself may facilitate increases in emotional granularity in depressed individuals, such that it may serve both empirical and interventional functions. We replicated and extended these findings in healthy adults, using data from an intensive ambulatory assessment study including experience sampling, peripheral physiological monitoring, and end-of-day diaries. We also identified factors that might distinguish individuals who showed larger increases over the course of experience sampling and examined the extent of the impact of these factors. We found that increases in emotional granularity over time were facilitated by methodological factors, such as number of experience sampling prompts responded to per day, as well as individual factors, such as resting respiratory sinus arrhythmia. These results provide support for the use of experience sampling methods to improve emotional granularity, raise questions about the boundary conditions of this effect, and have implications for the conceptualization of emotional granularity and its relationship with emotional health.

## Introduction

Individuals differ in their ability to create instances of emotion that are precise and context-specific – a skill referred to as *emotional granularity* (Tugade et al., 2004) or *emotion differentiation* (Barrett et al., 2001). The construct of emotional granularity highlights emotional experiences that are differentiated based on current or anticipated circumstances. As typically measured, emotional granularity represents the extent to which an individual distinguishes between like-valenced emotions (e.g., anger vs. sadness and excitement vs. pride) over time. Recent studies have shown that emotional granularity varies not only between but also within individuals over time (Tomko et al., 2015; Erbas et al., 2018, 2021), suggesting that it may be shaped and even improved. Moreover, accumulating evidence illustrates that higher emotional granularity is often associated with positive health outcomes in both clinical and non-clinical samples (Kashdan et al., 2015; Smidt and Suvak, 2015; Barrett, 2017a; O’Toole et al., 2020; Thompson et al., 2021). To date, however, only a few studies have examined whether and how emotional granularity might be increased (e.g., Van der Gucht et al., 2019; Widdershoven et al., 2019). The answers to these questions are critical for gaining a fuller understanding of the nature of the construct (e.g., its stability over time) as well as for charting its translational potential. In this paper, we provide an initial answer to these questions by first assessing change in participants’ emotional granularity across a two-week intensive ambulatory assessment study including experience sampling, peripheral physiological monitoring, and end-of-day diaries. We then explore the relationship between within-individual change and a set of potentially influential methodological and individual factors.

### Conceptualizing Emotional Granularity

Emotional granularity is one of multiple related constructs for individual differences in the experience of emotion and has similarities and differences with each. For example, emotional granularity has been described as a type or facet of emotional complexity (Kang and Shaver, 2004; Lindquist and Barrett, 2008; Grühn et al., 2013; O’Toole et al., 2020). Whereas emotional complexity can also refer to the simultaneous experience of multiple emotions or the variability or range of emotional experiences (Kang and Shaver, 2004; Grühn et al., 2013), emotional granularity refers specifically to the precision of emotional experience (Lindquist and Barrett, 2008). Accordingly, emotional granularity has been considered equivalent to (aspects of) emotional clarity (Boden et al., 2013; Cameron et al., 2013) and emotional awareness (Cameron et al., 2013; Mankus et al., 2016), as these constructs also require that individuals unambiguously identify and describe their experienced emotions. The construct of alexithymia describes the inability to identify and describe emotional experiences, and thus is inversely related to emotional granularity (Bermond et al., 1999; Edwards and Wupperman, 2017), with alexithymia equivalent to very low emotional granularity. Where emotional granularity most differs from complexity, clarity, awareness, and alexithymia (among others), however, is in its emphasis on context-specificity.

The central idea behind granularity is that emotional experiences are most adaptive when they are tailored to the needs of the situation at hand. This idea has been elaborated within constructionist, functionalist, and appraisal-based accounts of emotion (O’Toole et al., 2020). Constructionist accounts, such as the theory of constructed emotion (Barrett, 2006, 2012, 2013, 2017a,b), propose that the brain uses prior experience (i.e., concepts) to make meaning of the current situation and issue predictions about what is likely to occur next. The experience of emotion occurs when the brain issues a prediction using a concept for emotion. More context-specific predictions – which come from more precise (emotion) concepts – are more efficient because they better anticipate probable actions and upcoming energy needs (Hoemann et al., 2021). Functionalist (Goldston et al., 1992; Shiota et al., 2014; Plonsker et al., 2017) and appraisal-based accounts (Boden et al., 2013; Erbas et al., 2014, 2018, 2021; Thompson et al., 2021) of emotional granularity, in turn, hypothesize that differentiated emotional experiences are adaptive because they provide more specific or accurate information about the current situation, which enables individuals to react appropriately and engage in more effective emotion regulation (Kalokerinos et al., 2019).

To study the context-specific precision of emotional granularity, scientists need situated emotional experience data that are obtained across multiple contexts. These data are most commonly collected using experience sampling methods (Csikszentmihalyi and Larson, 1987) or ecological momentary assessment (Stone and Shiffman, 1994; see also Thompson et al., 2021), in which participants are prompted to report on multiple emotional experiences per day over the course of multiple days (e.g., Tugade et al., 2004). In principle, this measurement approach allows researchers to examine patterns of responses over time using real-world events. In practice, however, a participant’s emotional granularity is usually operationalized as the extent to which their intensity ratings for various emotion adjectives covary across all assessments [e.g., using an intra-class correlation (ICC; Shrout and Fleiss, 1979)], thereby representing an aggregate (i.e., trait) estimate of granularity. Recently, researchers have begun to estimate granularity at the momentary and/or day level (Tomko et al., 2015; Grossmann et al., 2016; Erbas et al., 2018, 2021) and have found that lower emotional granularity within individuals is predictive of behavioral tendencies (e.g., self-reported impulsivity; Tomko et al., 2015) and predicted by current distress and negative affect (Erbas et al., 2018). Furthermore, recent studies have leveraged this within-person variability to examine whether emotional granularity can be increased. For example, Van der Gucht et al. (2019) showed that a mindfulness-based intervention led to improvements in granularity both immediately following the intervention and at a follow-up assessment several months later. The possibility of increasing emotional granularity *via* intervention becomes especially relevant when considering the relationship between higher emotional granularity and various positive mental, behavioral, and social outcomes.

### Associations With Positive Outcomes

Reviews and meta-analyses describe a generally positive relationship between emotional granularity and health outcomes (Kashdan et al., 2015; Smidt and Suvak, 2015; Barrett, 2017a; O’Toole et al., 2020; Thompson et al., 2021). Briefly, individuals higher in emotional granularity are less likely to be diagnosed with a range of mental disorders (e.g., Frewen et al., 2008; Suvak et al., 2011; Erbas et al., 2013; Selby et al., 2013; Kimhy et al., 2014), including depression (Demiralp et al., 2012) and anxiety disorders (Mennin et al., 2005; Kashdan and Farmer, 2014). Higher granularity in non-clinical samples is also related to fewer symptoms associated with depression (Erbas et al., 2014, 2018; Starr et al., 2017; Willroth et al., 2019) and anxiety (Mennin et al., 2005; Seah et al., 2020). Correspondingly, higher granularity is linked to healthier coping behaviors. Individuals with higher granularity report less alcohol consumption during negative emotional experiences (Kashdan et al., 2010), fewer urges to binge eat (Dixon-Gordon et al., 2014), and lower incidence of drug relapse (Anand et al., 2017). Higher emotional granularity also results in fewer negative social outcomes, including decreased urges to physically aggress when provoked (Pond et al., 2012), and reduced neural responses to social rejection (Kashdan et al., 2014). These positive outcomes are more consistently associated with emotional granularity for negative emotions than for positive emotions (O’Toole et al., 2020; Thompson et al., 2021). Nevertheless, there is evidence that higher positive emotional granularity is linked to greater psychological resilience (Tugade et al., 2004).

In addition, increases in emotional granularity, writ broadly, appear to covary with improvements in mental health and other positive outcomes over time. Putting feelings into specific words has been shown to enhance psychotherapeutic efficacy (Kircanski et al., 2012), whereas the inability to do so (i.e., alexithymia) is a negative predictor of success across many disorders (Samur et al., 2013). In a prospective study of individuals with major depressive disorder, those whose alexithymia decreased over the course of a year were more likely to have reduced depressive symptoms (Honkalampi et al., 2001). Emotion-related training in children and adolescents resulted in better self-regulation, social functioning, and academic performance (Hagelskamp et al., 2013; Rivers et al., 2013). In adults, brief emotional granularity training has been shown to improve participants’ ability to make nuanced distinctions between emotions and to better understand how their emotions impacted judgments (Cameron et al., 2013). Finally, and compellingly, a recent study by Widdershoven et al. (2019) demonstrated that experience sampling improved emotional granularity in depressed individuals – effectively suggesting that this common method of assessment may serve both empirical and interventional functions (see also Myin-Germeys et al., 2016). However, it is not yet known whether the benefits of experience sampling or other forms of ambulatory assessment (Trull and Ebner-Priemer, 2013) may extend to non-clinical samples, or whether certain methodological and individual factors may facilitate increases in granularity over time.

### The Present Study

In the present study, we sought to replicate and extend the findings from Widdershoven et al. (2019) in a non-clinical sample. To do so, we used existing data from an intensive ambulatory assessment study including experience sampling, peripheral physiological monitoring, and end-of-day diaries (Hoemann et al., 2020a, 2021). Participants completed approximately 14, 8-h days of ambulatory assessment, during which their electrocardiogram (ECG), impedance cardiogram (ICG), electrodermal activity, movement, and posture were recorded. Participants responded to experience sampling prompts in the moment, and then elaborated on these responses in end-of-day diaries. As part of these diary entries, participants rated their experience of each event on a set of 18 emotion adjectives and described what was happening and how they were feeling at the time they received each prompt. This data set provided us with the opportunity to test for change in emotional granularity across a longer ambulatory assessment period than used in other studies (e.g., Erbas et al., 2018; Van der Gucht et al., 2019; Widdershoven et al., 2019). This data set also provided a unique opportunity to investigate a range of methodological, behavioral, and physiological variables that may facilitate increases in emotional granularity over time.

Based on the prior literature, we identified seven factors that might distinguish individuals who showed larger increases in granularity over the course of ambulatory assessment. Four of these were “methodological” factors, in that they were directly related to participants’ engagement with the study protocol. The first two factors were the number of ambulatory assessment days completed by each participant and the mean number of experience sampling prompts responded to each day – the latter of which provided for a “dose-response” analysis (following Widdershoven et al., 2019). The third and fourth factors were derived from the event descriptions participants provided in the end-of-day diaries: the mean length of these entries and the mean percentage of affective language used in these entries. These factors were motivated, respectively, by evidence linking expressive writing (e.g., Pennebaker and Chung, 2011) and affect labeling (e.g., Torre and Lieberman, 2018) to positive health outcomes. Writing longer event descriptions may reflect more time spent attending to daily emotional events and may also facilitate the formation of more coherent narratives about these events (see also Burton and King, 2004; Baikie and Wilhelm, 2005). Similarly, using more affective language (including emotion words) to describe experience may reflect increased emotional awareness and meaning-making (Lane et al., 1990; Ottenstein and Lischetzke, 2019).

The final three factors we identified were “individual” factors, which reflected differences in participants’ affective experience and peripheral physiological activity that were not directly related to the study protocol. Two of these factors were participants’ mean self-reported positive and negative affect (following Van der Gucht et al., 2019). Prior work has shown that differences in affect are related to differences in emotional granularity both within (Erbas et al., 2018) and across individuals (e.g., Demiralp et al., 2012), and that measures of mean affect are strongly predictive of psychological health (Dejonckheere et al., 2019). Lastly, to examine the potential relationship between emotional granularity and peripheral physiological activity, we included resting respiratory sinus arrhythmia (RSA) as our seventh factor. RSA is the variation in heart rate due to respiration and is typically measured as heart rate variability occurring within a specific respiratory frequency range (0.12–0.40 Hz), which is an estimate of vagal (i.e., parasympathetic) influence on the heart (Berntson et al., 1993, 1997; Task Force European Society of Cardiology, 1996). Previous research suggests that higher resting RSA is associated with better emotional and mental health (for a review, see, e.g., Balzarotti et al., 2017) and may facilitate emotional learning (e.g., Pappens et al., 2014).

Using these data, we assessed change in emotional granularity over the course of ambulatory assessment using person-specific regression analyses. These regressions estimated, for each participant, the relationship between assessment day (e.g., day 1 and day 2) and daily values for positive and negative emotional granularity. We conducted separate analyses by valence based on the prior literature showing differential benefits of positive versus negative granularity (Thompson et al., 2021) as well as differential change over time (Widdershoven et al., 2019). We first tested for overall (i.e., group level) change in emotional granularity by comparing the resulting regression coefficients (i.e., slopes) against a null hypothesis of no change. We predicted that both positive and negative granularity would progressively increase over time. Then, in exploratory analyses, we entered the slopes as dependent variables in Bayesian multiple linear regressions including the seven selected methodological and individual factors. This approach allowed us to assess the evidence for these factors’ influence on any increase in emotional granularity over time.

## Materials and Methods

The data used in the present study were collected as part of a larger study on affective experience and decision making in daily life and were previously reported in Hoemann et al. (2020b, 2021). All experimental protocols described below were approved by the Northeastern University Institutional Review Board (IRB# 16-01-13). These methods were carried out in accordance with the relevant guidelines and regulations for research with human subjects.

### Participants

Sixty-seven participants ranging in age from 18 to 36 years (55% female; 38.8% White, 3.0% Black, 29.8% Asian, and 28.4% other; *M* = 22.8 years, *SD* = 4.4 years) were recruited from the greater Boston area through posted advertisements, and Northeastern University classrooms and online portals. Eligible participants were non-smoking, fluent English-speakers, and were excluded if they had a history of cardiovascular illness or stroke, chronic medical conditions, mental illness, asthma, skin allergies, or sensitive skin. Eligible participants also confirmed they were not taking medications known to influence autonomic physiology including those for attentional disorders, insomnia, anxiety, hypertension, rheumatoid arthritis, epilepsy/seizures, cold/flu, or fever/allergies. Informed consent was obtained from all participants before beginning the study. Participants received $490 as compensation for completing all parts of the study, plus up to $55 in compliance and task incentives (for details, see page 1 of the Supplementary Material).

Of the 67 recruited participants, six withdrew and an additional nine were dismissed due to poor compliance with scheduling and prompt response requirements, as detailed below. Fifty-two participants completed ambulatory assessment, with two participants excluded because they did not complete the full study protocol including an in-lab session after the ambulatory assessment. The final sample size was 50 (54% female; 40% White, 2% Black, 44% Asian, and 14% other; *M* = 22.5 years, *SD* = 4.4 years). A sensitivity analysis in G*Power (version 3.1) confirmed that this data set was large enough to detect a difference from a constant (i.e., a one-sample *t*-test) with a medium effect size (*d* = 0.40–0.50), assuming *α* < 0.05 and power (1 − *β*) > 0.80.

### Procedure

Participants completed approximately 14 days (*M* = 14.4, *SD* = 0.6) of ambulatory assessment distributed across a three- to four-week period (*M* = 24.9 days, *SD* = 5.5 days). The study protocol included experience sampling with peripheral physiological monitoring, as well as end-of-day diaries, which enabled more comprehensive modeling of affective experience. Importantly, we also implemented a novel physiologically triggered experience sampling procedure, as described below, which enabled more efficient sampling of psychologically salient moments. Before and after the ambulatory assessment protocol, participants attended two in-lab sessions, in which they completed tasks and questionnaires that are not reported here (for an overview, see Hoemann, et al., 2020a).

Participants scheduled assessment days in advance according to their schedule, excluding weekends, within the allotted period. As such, not all assessment days occurred consecutively. On each day of ambulatory assessment, participants came to the laboratory to be outfitted with the peripheral physiological monitoring equipment. These sessions typically occurred between 8 and 9 am but varied between 7:30 am and 2:30 pm according to participants’ schedules. Participants could not begin without functioning monitoring equipment, and so did not complete the daily protocol if they did not attend the session to be instrumented. In the event, a participant was unable to make a scheduled session, or the equipment was not functioning properly, the assessment day was rescheduled. In principle, the protocol was for 14 assessment days. If there were pervasive issues with the physiological monitoring equipment, participants were requested to complete (and compensated for) additional assessment days. To limit attrition, participants were retained in the study if they completed at least 12 days of ambulatory assessment with usable data. Participants who were unable to complete the minimum number of days within a four-week period were dismissed from the study.

Participants were outfitted with sensors and portable equipment to measure their ECG, ICG, EDA, and bodily movement and posture (*via* accelerometers). All physiological measures were recorded on a mobile impedance cardiograph from the MindWare Technologies LTD (Model # 50-2303-02, Westerville, OH), which participants wore clipped to their clothing on the hip. The cardiograph also collected continuous three-axis accelerometry data that were used to assess movement. Participants wore two inertial measurement units (IMUs) from LP-Research (Minato-ku, Tokyo, Japan) to derive measures of posture and changes in posture. One IMU was placed medially on the sternum and the other IMU was placed on the front of the thigh. See page 1 of the Supplementary Material for additional acquisition details. Participants were instructed to continue physiological recordings for 8 h each day, after which they could remove and recharge all equipment. Participants did not remove sensors until the end of each experience sampling day, unless instructed by the experimenters (e.g., due to equipment issues).

Physiological and accelerometric data were recorded continuously throughout the day and the recording devices communicated *via* Bluetooth to a Motorola Moto G4 smartphone. A custom smartphone application, MESA (MindWare Technologies LTD, Westerville, OH), processed the ECG and accelerometer data in real time, and initiated an experience-sampling prompt anytime a substantial, sustained change in interbeat interval (IBI; also known as heart period) was detected in the absence of movement or posture change, with a minimum interval of 5 min between prompts. Minimal movement was operationalized as any time none of the three accelerometry channels from the cardiograph (alone or in aggregate) exceeded a threshold of 10 cm/s^2^ within the preceding 30 s. Absence of posture change was operationalized as any time when the relative orientation of the IMUs did not change within the preceding 30 s. On the first day of sampling, a substantial, sustained change in IBI was operationalized as a change of more than ±167 ms for at least an 8-s period. On subsequent days, this IBI parameter was manually adjusted to ensure each participant received approximately 20 prompts per day. This number of prompts was intended to ensure that participant had sufficient opportunities to respond, given that we could not guarantee the exact number of prompts that would be physiologically triggered.

Ultimately, participants received an average of 21.57 (*SD* = 6.06) prompts per day. The total number included an average of two “random” prompts each day. These prompts occurred in the absence of movement or posture change but were not contingent on a change in IBI. Random prompts were spread throughout the assessment day, such that one was sent in the first 4 h and one in the second 4 h. Participants were informed that they did not have to respond to all the prompts they received throughout a given day; they reported liking that they could be flexible in choosing when to respond with meaningful information. On average, participants responded to a prompt every 54 (*SD* = 13) min.

To remain in the study, participants were required to respond to a minimum of three prompts each day. In addition, for the purposes of incentivizing participation and limiting attrition, the ambulatory assessment protocol was broken into three pay periods (days 1–5, 6–10, and 11–14). Participants were required to respond to an average of at least six prompts per day during each period to remain in the study and received a bonus payment for each pay period where they completed an average of eight prompts per day (for details, see page 1 of the Supplementary Material). Compliance was assessed during instrumentation sessions: Experimenters would review participants’ data from the prior assessment day and discuss any questions or concerns. Participants ultimately responded to an average of 8.65 prompts (*SD* = 1.09) per day, consistent with prior experience sampling studies that have asked participants to respond to 10 prompts per day (e.g., Tugade et al., 2004; Widdershoven et al., 2019). Days in which participants responded to more than 10 prompts were uncommon (19% of participant days) and those in which they responded to more than 15 prompts were rare (4% of participant days).

At each sampling event (regardless of whether it was physiologically or randomly triggered), participants were prompted to respond to a series of questions presented in the MESA phone application. These data were not analyzed in the present study but are summarized here to be transparent about all elements of the ambulatory assessment protocol. First, participants provided a brief free-text description of what was going on at the time they received the prompt. Second, participants rated their current valence and arousal, each on a 100-point continuous slider scale ranging from −50 (very unpleasant or deactivated) to +50 (very pleasant or activated). Third, participants provided a brief free-text description of their social context by: writing “alone,” listing the initials of direct interaction partners, and/or writing “group” (to indicate the presence of a large number of other people). Fourth, participants selected a major activity from a drop-down list consisting of: “socializing,” “eating,” “exercising,” “watching TV,” “working,” “commuting,” “using computer/email/Internet,” “preparing food,” “on the phone,” “praying/meditating/worship,” “napping,” “taking care of children,” “housework,” or “other.” Fifth, participants self-generated words to label their current affective experience. Participants were able to provide as many words as they felt necessary but were required to input at least one. For each self-generated word, participants were asked to provide an intensity rating on a Likert-style scale from 1 (“not at all”) to 5 (“very much”). Finally, participants received one of two possible single-item decision tasks: either a temporal discounting problem or a scrambled anagram problem.

Immediately upon finishing each day, participants automatically received a modified day reconstruction diary (Kahneman et al., 2004) *via* SurveyMonkey (San Mateo, CA). Participants were requested to complete the diary as soon as possible after finishing their day of experience sampling. In this diary, they were presented with some of the information provided for each prompt during the day: the event time, brief description, social context, and major activity. Of note, participants were not presented with the words they had self-generated to label their current affective experience. Using this information as a guide, participants were asked to provide additional details about each experience sampling event. First, they were asked to describe the social context of the event, including a brief description of any initials (e.g., “SB is a coworker”). Second, they were asked to provide a description of what was happening as they received the prompt. Participants were requested to choose three sampling events for which they provided a longer description (>200 words). Only three detailed descriptions were requested to limit the amount of burden imposed, as determined through pilot testing. Next, they were asked to recall their affective experience at the time of the prompt in two ways: (1) using slider scales to rate their valence and arousal and (2) using Likert-style scales from 0 (“not at all”) to 6 (“very much”) to rate their experienced intensity on a standard set of 18 emotion adjectives (“afraid,” “amused,” “angry,” “bored,” “calm,” “disgusted,” “embarrassed,” “excited,” “frustrated,” “grateful,” “happy,” “neutral,” “proud,” “relieved,” “sad,” “serene,” “surprised,” and “worn out”). These standard intensity ratings were requested in the end-of-day diary, rather than at each experience sampling prompt, to reduce participant burden in the moment. Lastly, participants were asked to respond to a series of seven descriptive appraisal questions developed based on the Geneva Appraisal Questionnaire (Geneva Emotion Research Group, 2002). End-of-day diary data regarding events’ social context, associated valence and arousal ratings, and appraisals were not analyzed in the present study but are mentioned with transparency in mind.

### Data Preparation

We computed estimates of daily emotional granularity from the intensity ratings for the 18 emotion adjectives rated in the end-of-day diaries. Data from late diaries (i.e., completed the following day) were excluded from analysis (4% of participant days). Following prior literature (e.g., Tugade et al., 2004), we estimated granularity as an ICC using agreement with averaged raters (“A-k” method; Shrout and Fleiss, 1979). Higher ICC values reflected lower emotional granularity (i.e., greater shared variance among adjectives’ ratings). To ensure reliable day-level ICCs (Erbas et al., 2018), we excluded days when participants responded to fewer than six prompts (8% of participant days).^1^ Similarly, because negative ICC values are beyond the theoretical range, they were also excluded from analysis (following, e.g., Erbas et al., 2018).^2^ We computed separate indices of daily granularity for pleasant (positive) versus unpleasant (negative) emotions, with this distinction based on normative ratings (Warriner et al., 2013). ICCs were Fisher *r*-to-*z* transformed to fit the variable to a normal probability distribution. We multiplied these transformed values by −1 to yield estimates of daily granularity that scaled intuitively, such that lower (more negative) values reflected lower granularity, and higher (less negative) values reflected higher granularity.

For each participant, we also computed a set of seven predictor variables. Six of these were derived from the end-of-day diary data. First, we counted the number of days completed by each participant and calculated the mean number of experience sampling prompts responded to each day. Next, we entered the event descriptions participants provided in the end-of-day diaries into the Linguistic Inquiry and Word Count software (LIWC; Pennebaker et al., 2015) and used this to calculate the mean length of entries and the mean percentage of affective language used (i.e., from the LIWC “affect” dictionary). Using the intensity ratings for the 18 emotion adjectives, we calculated participants’ mean self-reported positive and negative affect as the average ratings of like-valenced emotion adjectives (with this distinction again based on normative ratings; Warriner et al., 2013).

The seventh predictor variable, resting RSA, was derived from the ambulatory peripheral physiological data. As reported in Hoemann et al. (2021), we first identified periods of seated rest in the ECG signal according to the following criteria: participant position was seated and not moving (i.e., no forward acceleration); participant maintained this position for at least 60 s and no experience-sampling prompt was triggered. We excluded data from the first 30 s of each period of seated rest to allow for the ECG signal to stabilize following movement. The ECG signal was processed following prior work (Hoemann et al., 2020a, 2021) using an in-house pipeline coded in Python. For each seated rest period, we derived resting RSA using 30-s bins and computed the mean across all bins. For each participant, we then took the grand mean across all seated rest periods. See page 1 of the Supplementary Material for additional details.

### Analysis

To assess change in emotional granularity over the course of ambulatory assessment, we conducted person-specific regression analyses estimating the relationship between assessment day (reflecting “time in study”) and daily granularity (i.e., inverse day-level ICCs). We fit two models for each participant: one in which assessment day predicted daily granularity for positive emotions and one in which assessment day predicted daily granularity for negative emotions. All variables were standardized prior to analysis for interpretability and comparability across participants. We operationalized change in granularity as the regression coefficient associated with assessment day (i.e., the slope of the independent variable). Positive slopes, then, indicate an increase in emotional granularity over time, whereas negative slopes indicate a decrease. We assessed group-level change in emotional granularity using separate one-sample *t*-tests, in which the slopes for positive and negative granularity were compared to zero, and we estimated the effect size of this change from the corresponding Cohen’s *d* value.

In general, change over time can be modeled using mixed-effect approaches or using latent-curve approaches (for discussion, see McNeish and Matta, 2018). We broadly followed a mixed-effect approach because our models were simple, each with one outcome variable (i.e., positive versus negative emotional granularity), and because this approach can more easily accommodate small samples and missing data (McNeish and Matta, 2018). In principle, we could have used a mixed-effect approach to estimate the mean change in granularity (i.e., fixed effect) for the entire sample, along with random effects capturing each participant’s deviation from the mean change. However, these random effects would not have allowed us to assess the relationship between each participant’s absolute change in emotional granularity and individual differences in affective experience and engagement with the study protocol. To do this, we entered the slopes for positive and negative granularity as the dependent variables in separate multiple linear regressions with the seven selected factors as predictors. All variables were again standardized prior to analysis for interpretability. These regressions were fit with Bayesian estimation to quantify evidence in favor of or against any factor’s relationship to an increase in granularity (Wagenmakers et al., 2018).

## Results

Both positive and negative emotional granularity increased over the course of ambulatory assessment: positive *t*(49) = 3.54,*p* < 0.001, two-tailed; negative *t*(49) = 2.26, *p* ≤ 0.03, two-tailed. That is, the shared variance among participants’ emotion intensity ratings decreased with more time in study.^3^ The estimated effect sizes – *d* = 0.50 and *d* = 0.32, respectively – indicated that experience sampling had a medium treatment effect and that this was larger for positive than negative granularity. Nevertheless, the direction and magnitude of change in emotional granularity varied across the sample (see Supplementary Figures 1, 2 for plots across individual participants). Participants also differed in terms of engagement in the study protocol, affective experience, and peripheral physiological activity, as represented by the predictor variables. Descriptive statistics for all variables are provided in Table 1.

**Table 1:** Descriptive statistics across individuals.

Variable	Mean	*SD*	Min	Max
**Dependent variables**				
Change in positive emotional granularity	0.16	0.33	−0.54	0.92
Change in negative emotional granularity	0.12	0.37	−0.70	0.97
**Predictor variables**				
Included assessment days	13.12	1.41	8	15
Mean number of prompts per day	8.98	1.14	6.86	12.31
Mean length (words) of event descriptions	94.16	17.23	65.29	137.76
Mean percentage of affective language	5.39	1.51	2.41	9.31
Mean positive affect (0–6 scale)	2.22	0.63	1.49	4.41
Mean negative affect (0–6 scale)	1.62	0.42	1.16	3.25
Mean resting RSA (natural log; ln)	8.82	0.80	6.51	10.26

Daily emotional granularity estimated as the inverse of the intra-class correlation over the intensity ratings for positively- and negatively-valenced emotion adjectives, respectively. Change in positive and negative emotional granularity operationalized as the regression coefficient associated with assessment day when used to predict daily granularity, such that positive values reflect increases in granularity over time and negative values reflect decreases in granularity over time.

**Figure 1:**
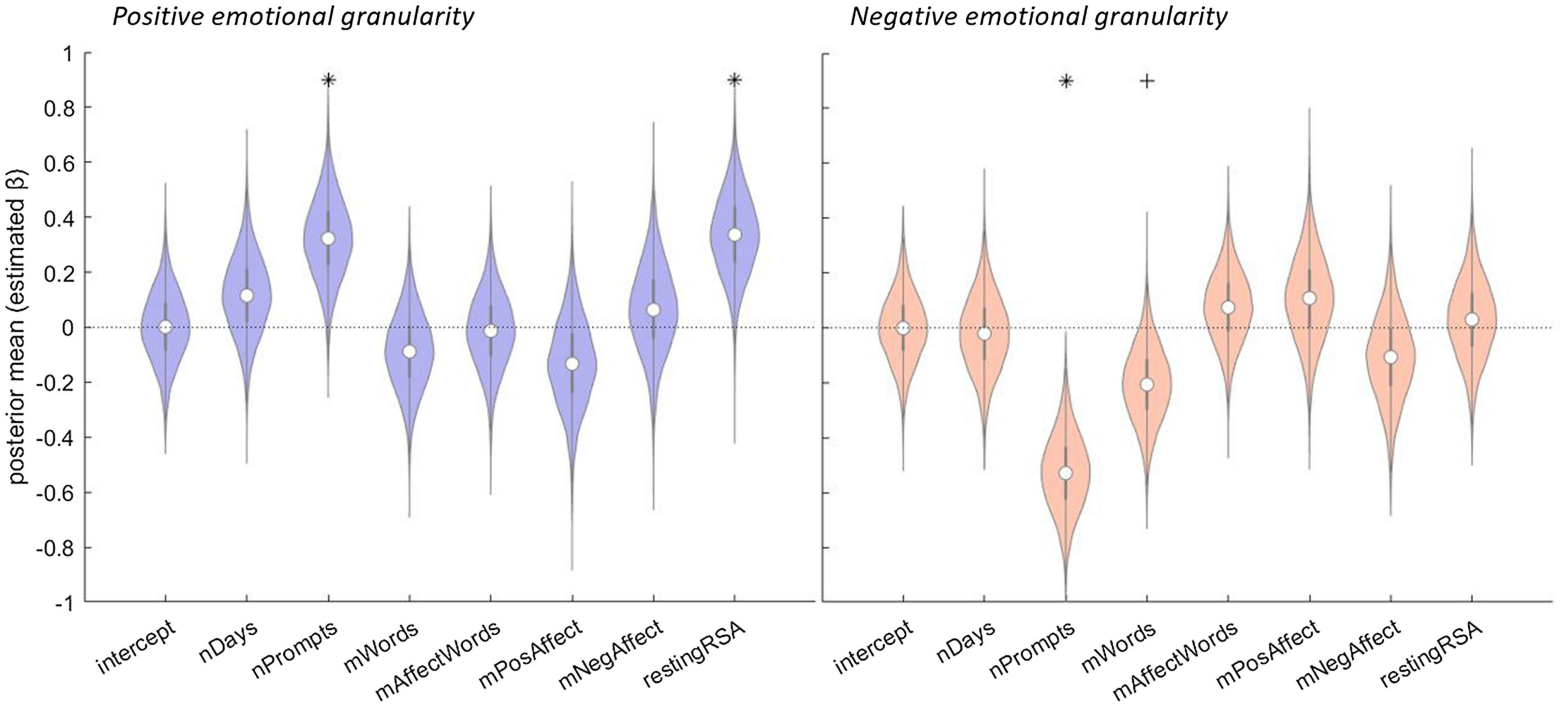
The relationship between methodological and individual factors and change in emotional granularity over the course of ambulatory assessment. *Left panel:* positive granularity as the dependent variable. *Right panel:* negative granularity as the dependent variable. The results of Bayesian multiple regressions are shown. Violins represent the posterior distributions (i.e., estimated *β*s) for each factor based on 10,000 Monte Carlo samples. In the boxplots, the center dots represent medians, and the thick gray lines extend to the first and third quartiles. The whiskers extend from the quartile to 1.5 times the interquartile range. The likelihood of a factor’s relationship to change in emotional granularity (i.e., its posterior probability) is represented by the violins’ extents around zero, with zero indicated by the dotted horizontal black line. Factor names: nDays, number of days of ambulatory assessment included; nPrompts, mean number of experience sampling prompts responded to per day; mWords, mean length of event descriptions provided in end-of-day diaries; mAffectWords, mean percentage of affective language used in event descriptions; mPosAffect, mean self-reported positive affect; mNegAffect, mean self-reported negative affect; and restingRSA, mean respiratory sinus arrhythmia value across all periods of ambulatory seated rest. ^*^probability ≥ 95%; + probability ≥ 90%.

Figure 1 depicts the results of the regressions for increases in positive and negative emotional granularity, respectively, as predicted by the methodological and individual factors (for details, see Supplementary Tables 2 and 3). Each panel is a violin plot of the posterior distributions (i.e., estimated *β*s) for the intercept and all seven factors. The likelihood of a factor’s relationship to change in emotional granularity (i.e., its posterior probability) is represented by the extent to which each distribution (i.e., violin) overlaps zero (Franke and Roettger, 2019). For example, violins above zero with ≤ 5% of their area extending below zero represent factors that positively influence change in granularity with ≥ 95% probability, whereas violins below zero with ≤ 5% of their area extending above zero represent factors that negatively influence change in granularity with ≥ 95% probability.

As illustrated in the left panel of Figure 1, there is a 98% likelihood that the mean number of experience sampling prompts per day was positively associated with increase in positive emotional granularity [*β* = 0.32, 95% Credible Interval (CI) = (0.04, 0.61)], such that participants who responded to more prompts showed larger granularity increases over the course of ambulatory assessment. There is also a 99% likelihood that resting RSA was positively associated with an increase in positive emotional granularity [*β* = 0.34, 95% CI = (0.05, 0.53)], such that participants with higher seated resting RSA values showed larger granularity increases over the course of ambulatory assessment.^4^ There is no evidence that increase in positive emotional granularity was associated with the number of days of ambulatory assessment included [*β* = 0.11, 95% CI = (−0.18, 0.41)], the mean length of event descriptions provided in end-of-day diaries [*β* = −0.09, 95% CI = (−0.37, 0.20)], the mean percentage of affective language used in event descriptions [*β* = −0.02, 95% CI = (−0.30, 0.26)], or mean self-reported positive [*β* = −0.13, 95% CI = (−0.46, 0.20)] or negative [*β* = 0.06, 95% CI = (−0.27, 0.40)] affect.

As illustrated in the right panel of Figure 1, there is a > 99% likelihood that the mean number of experience sampling prompts per day was negatively associated with an increase in negative emotional granularity [*β* = −0.53, 95% CI = (−0.80, −0.24)]. In other words, participants who responded to more prompts showed smaller increases (or even decreases) in negative granularity over the course of ambulatory assessment. There is also a 94% likelihood that the mean length of event descriptions was negatively associated with an increase in negative emotional granularity [*β* = −0.21, 95% CI = (−0.48, −0.07)], such that participants who wrote more about each event showed smaller increases (or even decreases) over the course of ambulatory assessment. There is no evidence that increase in negative emotional granularity was associated with the number of days of ambulatory assessment included [*β* = −0.02, 95% CI = (−0.31, 0.27)], the mean percentage of affective language used in event descriptions [*β* = 0.07, 95% CI = (−0.20, 0.35)], mean self-reported positive [*β* = 0.11, 95% CI = (−0.21, 0.43)] or negative [*β* = −0.11, 95% CI = (−0.43, 0.22)] affect, or resting RSA [*β* = 0.03, 95% CI = (−0.27, 0.33)].

## Discussion

In a sample of healthy adults, we found that both positive and negative emotional granularity increased over the course of an intensive, two-week ambulatory assessment study that included peripheral physiological monitoring and end-of-day diaries. Subsequent exploratory analyses suggested that methodological factors, such as engagement with the study protocol (i.e., number of experience sampling prompts responded to each day and length of end-of-day event descriptions), and individual factors, such as affective experience and peripheral physiological activity (i.e., resting RSA), were related to the observed effects, and differentially influenced changes in positive and negative granularity. These findings broadly replicate and extend recent work (Widdershoven et al., 2019), providing further support for the use of ambulatory assessment methods to improve emotional granularity. These findings also raise questions about the boundary conditions associated with increases in emotional granularity and have implications for the conceptualization of emotional granularity and its relationship with emotional health.

Experience sampling, the most common method used to obtain a measure of emotional granularity (Thompson et al., 2021), allows researchers to examine patterns of emotion adjective co-occurrence over time using real-world events. It also requires participants to attend repeatedly to their emotional experiences, thereby providing a context that may facilitate increases in emotional granularity. Indeed, it has been hypothesized that higher emotional granularity may reflect habitual processing of affective stimuli in a differentiated and more complex manner (Lee et al., 2017), and that emotional granularity may be increased by intentionally reflecting upon and diversifying affective experiences (Cameron et al., 2013, 2014; Barrett, 2017a). It is possible that ambulatory assessment protocols such as that used here assist in these endeavors by asking participants to observe their emotions more frequently and/or thoroughly than they would otherwise. Although we did not systematically assess participants’ experiences with the current protocol, we did gather anecdotal evidence during daily check-ins and at the final study debriefing. In these informal reports, many participants indicated feeling more aware of their emotions and more “mindful” after completing the ambulatory assessment protocol and found this beneficial. These comments are consistent with the idea that experience sampling may direct or focus attention on emotional experience.

Several study design choices can influence whether and by how much emotional granularity increases over the course of a given ambulatory assessment protocol. Studies can differ in terms of protocol length (i.e., number of days) and intensity (i.e., the number of experience sampling prompts per day, the average duration between prompts, and the number of emotion terms rated; Thompson et al., 2021; see also Kirtley et al., 2021). Two of these parameters – length of protocol and number of prompts administered – varied among participants in the current study and could be assessed for their impact. The results of our exploratory analyses suggest that the number of prompts responded to each day may be a key factor in the extent to which emotional granularity increases with time in study. We also found that this factor had the opposite effect on positive and negative emotional granularity. There was evidence that more prompts facilitated increases in positive granularity, suggesting a dose-response relationship, yet there was also evidence that more prompts reduced increases (or even facilitated decreases) in negative granularity. The effect on negative granularity in our results may be due to increased sampling fatigue which, by increasing distress or negative affect, could have reduced granularity for negative emotions (Erbas et al., 2018). However, recent work by Eisele et al. (2020) provides evidence against this possibility. That team found that longer prompts increased participant burden, whereas higher prompt frequency was not associated with negative consequences. Because we allowed participants to choose (beyond a necessary minimum) how many prompts they responded to, the effect of number of prompts may not be observable in studies that stipulate a specific number of prompts to be answered.

In addition, this study was the first to use a two-step approach to assess emotional granularity, in which participants received experience sampling prompts during the day and provided additional information (including emotion intensity ratings) for each prompt in end-of-day diaries. The combination of momentary and daily diary assessment has precedent in the literature (e.g., Businelle et al., 2016); it is also not uncommon to assess emotional granularity using daily diary methods in which participants rate emotional events from earlier in the day (e.g., Barrett et al., 2001; Dasch et al., 2010; for a review, see Thompson et al., 2021). It is possible that a two-step approach influenced the data obtained at end of day by, for example, providing an opportunity for initial emotion regulation in the moment (e.g., *via* affect labeling; Torre and Lieberman, 2018) or introducing some recall bias (e.g., Levine and Safer, 2002; but see Schneider et al., 2020). However, the end-of-day diaries provided participants with details recorded at the time of the experience sampling prompts, in theory allowing them to re-instantiate earlier experiences with greater fidelity. More broadly, a two-step approach raises the question of which aspect of ambulatory assessment was responsible for the observed increases (i.e., the events captured during the day versus the reflection on these events in the evening). Future studies can address these considerations by testing the effect of ambulatory assessment on healthy adults using a more typical experience sampling protocol (e.g., Tugade et al., 2004), without the addition of peripheral physiological monitoring or end-of-day diaries. In this respect, the present study design represents an upper bound of study complexity and cannot be used to determine the minimum necessary protocol elements that are needed to observe increases in emotional granularity.

In this study, we sought to replicate and extend recent work by Widdershoven et al. (2019). The authors of that study found that experience sampling improved both positive and negative emotional granularity in depressed individuals. In contrast to the present study, however, the change in positive granularity did not reach significance. Widdershoven et al. (2019) also did not find evidence of a dose-response relationship between number of experience sampling prompts and increase in granularity. Those authors attributed the latter result to a relatively small sample size (*N* = 55), yet a dose-response relationship was found in the present study, which was also limited by a similar sample size (*N* = 50). These differences in results could be due to differences in the method of ambulatory assessment used. For example, participants in the present study completed approximately 14 days of assessment across a three- to four-week period, whereas participants in Widdershoven et al. (2019) completed 18 days of experience sampling across a six-week period (at a rate of three consecutive days per week). Participants in Widdershoven et al. (2019) also completed separate baseline and post-intervention measures of emotional granularity, whereas in the present study, changes were assessed continuously over the course of the ambulatory assessment period. As noted previously, the present study was not expressly designed with replication in mind, and so it may not be representative of the kinds of parameter settings that may be used in more typical ambulatory assessment studies.

These considerations notwithstanding, the present findings have implications for the conceptualization of emotional granularity. Until fairly recently, emotional granularity has been operationalized as a trait, using a single aggregate estimate per person (Tugade et al., 2004). The observation that emotional granularity changes over the course of experience sampling is consistent with other evidence that documents meaningful within-person variability in granularity over time (Tomko et al., 2015; Grossmann et al., 2016; Erbas et al., 2018, 2021). In particular, our finding of increases in emotional granularity as a function of study engagement supports the hypothesis that granularity is a skill that can be acquired and improved (Kashdan et al., 2015). The idea that emotional granularity may be enhanced through practice has been advanced by constructionist accounts of emotion, which propose that intentional focus on emotional experience may help to elaborate and diversify emotion concepts (Barrett, 2017a; see also Averill, 1999; Hoemann, et al., 2020b). However, it is not yet clear what form this practice should take.

As discussed above, we found that participants who responded to more experience sampling prompts per day showed larger increases in positive emotional granularity but smaller increases (or even decreases) in negative emotional granularity. This finding may be related to differential effects of attending to positive versus negative experience. For example, attending to positive experiences more often or more deliberately may encourage savoring, or adaptive forms of rumination. This possibility is supported by prior studies that have instructed participants to reminisce about past experiences or focus on the present moment as a means of intensifying or prolonging positive feelings, with corresponding benefits for wellbeing (e.g., Smith et al., 2014). This possibility is further consistent with prior work linking positive granularity with the broaden-and-build framework for positive emotions and effective coping (Tugade et al., 2004). However, there is also evidence to suggest that higher positive granularity may impede savoring (Starr et al., 2017). Research is needed to further investigate the relationship between positive granularity and real-world outcomes, and the circumstances in which positive granularity is beneficial (for discussion, see Thompson et al., 2021).

In contrast, increased attention to negative experiences may encourage maladaptive forms of rumination. This possibility is supported by prior studies that have found associations between low negative granularity and rumination (e.g., Di Schiena et al., 2011; Starr et al., 2017). It is also supported by findings from the expressive writing literature suggesting that the use of negative emotion words is non-linearly related to improved wellbeing, such that a moderate number of negative emotion words is associated with greatest benefit (for review, see Pennebaker et al., 2003). Indeed, in the present study, we also found that participants who wrote longer event descriptions in the end-of-day diaries showed smaller increases in negative emotional granularity. Taken together, these findings suggest that, past a certain point, attending more often or at greater length to one’s negative emotional experiences may reduce the benefits of practice. This possibility, and the long-term effects of practice on both positive and negative emotional granularity, remains to be tested by future research.

The present findings also contribute to our understanding of the relationship between emotional granularity, affective experience, and peripheral physiological activity. Our finding that larger increases in positive emotional granularity were associated with higher resting RSA is especially noteworthy given prior evidence of a positive relationship between resting RSA and both emotional and mental health (e.g., Balzarotti et al., 2017), and the potential role of RSA in facilitating emotional learning (e.g., Pappens et al., 2014). Our finding is also consistent with studies demonstrating associations between higher resting RSA and stable positive affect (Oveis et al., 2009; Koval et al., 2013) and builds on recent work with this same sample showing a positive relationship between overall emotional granularity and resting RSA in daily life (Hoemann et al., 2021). The exact nature of the link between emotional granularity and resting RSA is an open question. Both emotional granularity (Barrett et al., 2001; Kalokerinos et al., 2019) and resting RSA (e.g., Appelhans and Luecken, 2006; Geisler et al., 2010; Williams et al., 2015; Mather and Thayer, 2018) are associated with better self-regulation and adaptive coping strategies. Positive emotional granularity, although receiving less attention than negative granularity (O’Toole et al., 2020), has specifically been linked to psychological resilience (Tugade et al., 2004). The hypothesized mechanisms underlying these connections vary depending on the theoretical framework used, with some emphasizing neurobiological pathways and dynamics of RSA (Thayer and Lane, 2000; Porges, 2007), some highlighting functional advantages of positive emotions (Fredrickson, 2001; Shiota et al., 2014), and others proposing more domain-general models of psychological and physiological regulation (Barrett, 2017b; Gianaros and Jennings, 2018). Although the present findings cannot directly address questions of mechanism, they inform future studies by suggesting that emotional granularity is amenable to the experimental manipulation necessary to gain insight into causality.

This study, like Widdershoven et al. (2019), was inspired by accumulating research that uses experience sampling and other ambulatory assessment methods as a form of mental health intervention [i.e., ecological momentary interventions (EMIs; e.g., Myin-Germeys et al., 2016, 2018)]. The causal paths by which emotional granularity and emotional or mental health are related are not yet known. However, emotional granularity is a compelling potential target for intervention given growing evidence of associations between higher emotional granularity and positive health outcomes (reviewed in Kashdan et al., 2015; Smidt and Suvak, 2015; Barrett, 2017a; O’Toole et al., 2020; Thompson et al., 2021), as well as conceptual links between higher granularity and adaptive situated functioning (e.g., Barrett, 2017a; Erbas et al., 2021; Thompson et al., 2021). In this study, we have shown that emotional granularity can be increased in the absence of explicit instructions, suggesting that intensive ambulatory assessment can increase both positive and negative emotional granularity, perhaps by shifting attention to emotional experience in daily life. These findings join others (Cameron et al., 2013; Van der Gucht et al., 2019; Widdershoven et al., 2019) in laying a foundation for a line of transformative research on how emotional granularity training may shape everyday emotional experiences, with the potential for positive impacts on wellbeing and health.

## Data Availability Statement

Publicly available data sets were analyzed in this study. This data can be found at https://osf.io/dk569/.

## Ethics Statement

The studies involving human participants were reviewed and approved by the Institutional Review Board, Northeastern University. The patients/participants provided their written informed consent to participate in this study.

## Author Contributions

KH assisted with data collection and pre-processing, analyzed the data, and wrote the manuscript. KH and KQ designed the analysis. All authors reviewed and revised the manuscript. All authors contributed to the article and approved the submitted version.

## Conflict of Interest

The authors declare that the research was conducted in the absence of any commercial or financial relationships that could be construed as a potential conflict of interest.

## Publisher’s Note

All claims expressed in this article are solely those of the authors and do not necessarily represent those of their affiliated organizations, or those of the publisher, the editors and the reviewers. Any product that may be evaluated in this article, or claim that may be made by its manufacturer, is not guaranteed or endorsed by the publisher.

## Footnotes

^1^The data retained for analysis represented days when participants responded to at least six prompts, which could be interpreted as reflecting relatively high engagement from participants. At the same time, a six-prompt threshold is in keeping with the fact that participants were required to respond to a rolling average of six prompts per day to remain in the study.

^2^The decision to exclude negative day-level ICC values from analysis did not impact most participants: 29 of 50 (58%) participants had no such values; 13 (26%) had only one negative ICC; seven (14%) had two negative ICCs; and only one (2%) had more than two negative ICCs. Excluding the individual with more than two negative ICCs did not substantively change the results.

^3^Recent studies suggest that emotional granularity might be indexed by the words used to label affective experience (e.g., Ottenstein and Lischetzke, 2019). Affect labeling is also related to emotion regulation (e.g., Torre and Lieberman, 2018). As such, it is possible that the number of words participants used to label their current experience at each experience sampling prompt was related to changes in emotional granularity over time. We observed that the number of words used per prompt/day decreased over time (mean person-specific slope = −0.21, *SD* = 0.35). However, we also observed that the total number of words each participant used across the study was positively correlated with their overall positive (*r* = 0.21) and negative (*r* = 0.23) emotional granularity. Given these observations, the number of words used per prompt/day may not have a straightforward relationship with changes in emotional granularity. A full test of this hypothesis, with appropriate control for other important covariates, awaits future research.

^4^Respiratory sinus arrhythmia (RSA) is the phenomenon of respiratory-modulated variation in the time interval between successive heart beats or the interbeat interval (IBI). In the present study, substantive and sustained changes in IBI were used to trigger the majority of experience sampling prompts, leading to the question of whether the relationship between IBI and RSA could influence our findings. We consider this possibility unlikely. We derived estimates of resting RSA for each participant based on periods of seated rest during which no experience sampling prompts were triggered. In this way, we separated IBI changes associated with specific, triggered prompts from ongoing, natural respiratory-related variation. More broadly, experience sampling prompts could be triggered by decreases or increases in IBI (cardiac acceleration or deceleration, respectively; prompts could also be random). Momentary changes in IBI may reflect sympathetic and/or parasympathetic effects on the heart, whereas resting RSA reflects overall parasympathetic influence. Thus, these variables have distinct physiological bases and would not be expected to directly predict one another, even if measured during the same periods.
