# Supplementary material for: Emotional Granularity Increases With Intensive Ambulatory Assessment: Methodological and Individual Factors Influence How Much
Source: Front Psychol. 2021 Jul 28;12:704125. doi: 10.3389/fpsyg.2021.704125 (PMC8355493; doi:10.3389/fpsyg.2021.704125)
Supplement: Supplementary file 1 [file Data_Sheet_1.docx]

***Supplementary Material***

**Payment**

Participants received $30 for their first in-lab session, $20 per day for the first five days of experience sampling, $30 per day for the second five days of experience sampling, and $40 per day for the final four days of experience sampling. Participants were incentivized to respond to an average of eight prompts per day during experience sampling and received a $10 bonus for every pay period in which they made this target (i.e., up to three times total). Lastly, participants received $50 for their second and final in-lab session. Participants also received a $25 bonus for completion of an in-lab temporal discounting task.

**Physiological Measurement**

ECG and ICG were obtained using pre-gelled ConMed (Westborough, MA) Cleartrace Ag/AgCl sensors, connected via wires to the mobile impedance cardiograph. Sensor sites were cleaned with alcohol and abraded lightly with gauze. ECG was obtained using a modified lead II configuration, with recording electrodes placed on the distal right collarbone and an inferior left rib, respectively, and a reference electrode placed on an inferior right rib. The ECG signal was acquired at 500 Hz using a low cutoff of 0.5 Hz and a high cutoff of 45 Hz. ICG was obtained using a four-spot electrode configuration (Qu et al., 1986). Two inner recording electrodes were placed on the front of the torso: one at the base of the neck at the top of the sternum, and a second at the bottom of the sternum over the xiphisternal junction. Two outer source electrodes were placed on the back along the midline approximately 4 cm above and below the inner recording electrodes. The source electrodes passed a 4 mA, 100 kHz alternating current across the thorax. Basal impedance (Z_0_) was acquired at 500 Hz using a low cutoff of 10 Hz. dZ/dt was acquired using a low cutoff of 0.5 Hz and a high cutoff of 45 Hz. Electrodermal activity (EDA) was also recorded over an unobtrusive neck placement, but data were not used due to difficulties in detecting and removing artifacts, as well as generally low mean skin conductance levels.

**Physiological Signal Processing**

Physiological signals were processed following previous work (Hoemann et al., 2020, 2021) using an in-house pipeline coded in Python. Raw ECG signal was passed through an elliptic bandpass filter to remove baseline and high frequency noise. Initial quality checks were then performed for each beat, checking for overall waveform shape, and acceptable minimum, maximum, and minimum-to-maximum values (Table S1). R-peak detection for ECG was performed using established methods (Hamilton, 2002) and implemented using the BioSPPy package (Carreiras et al., 2018). Mean IBI was then derived as the average R-R interval. Additional quality checks (Supplementary Table 1) were performed on each IBI series to ensure that values were within acceptable ranges (300-2000 ms), and that expected beat-to-beat differences were consistent with normal beats and unlikely to be artifacts (following established benchmarks; Berntson et al., 1990). ECG data failing any quality check were excluded from analysis. RSA was derived from the IBI series. These calculations were coded to mimic the processing steps of standard HRV analysis software (MindWare Technologies LTD, Westerville, Ohio), including cubic interpolation of beat-to-beat IBI, detrending to minimize non-stationarity, tapering using a Hamming window, and lastly, fast Fourier transformation (FFT). RSA was calculated as the natural log of the area under the power spectrogram from .12 to .40 Hz.

**Supplementary Table 1.** Hyperparameters for Electrocardiogram (ECG) Signal Processing

| Hyperparameter | Value | Reference Value* |
| --- | --- | --- |
| Length of signal for flatness check | 1.2*sampling frequency | 0.8*sampling frequency |
| Minimum standard deviation for flatness check | 1.00E-05 | 1.00E-05 |
| Length for minimum-maximum check | 1.2*sampling frequency | 1.2*sampling frequency |
| Minimum/maximum for max/min check | -.005/.005 | -.005/.005 |
| Length of signal for skewness check | 1.2*sampling frequency | 1.2*sampling frequency |
| Length of sub-chunks for skewness check | .064*sampling frequency | .032*sampling frequency |
| Minimum skewness | .45 | .45 |

Note: *Taken from Nabian et al. (2018). Default parameters, originally selected based on data collected in laboratory settings, were found to be too aggressive for this ambulatory data set, so we relaxed these thresholds based on manual review of the results by experts (e.g., K.S.Q.) for a random subset of the data.

**Change in Emotional Granularity**


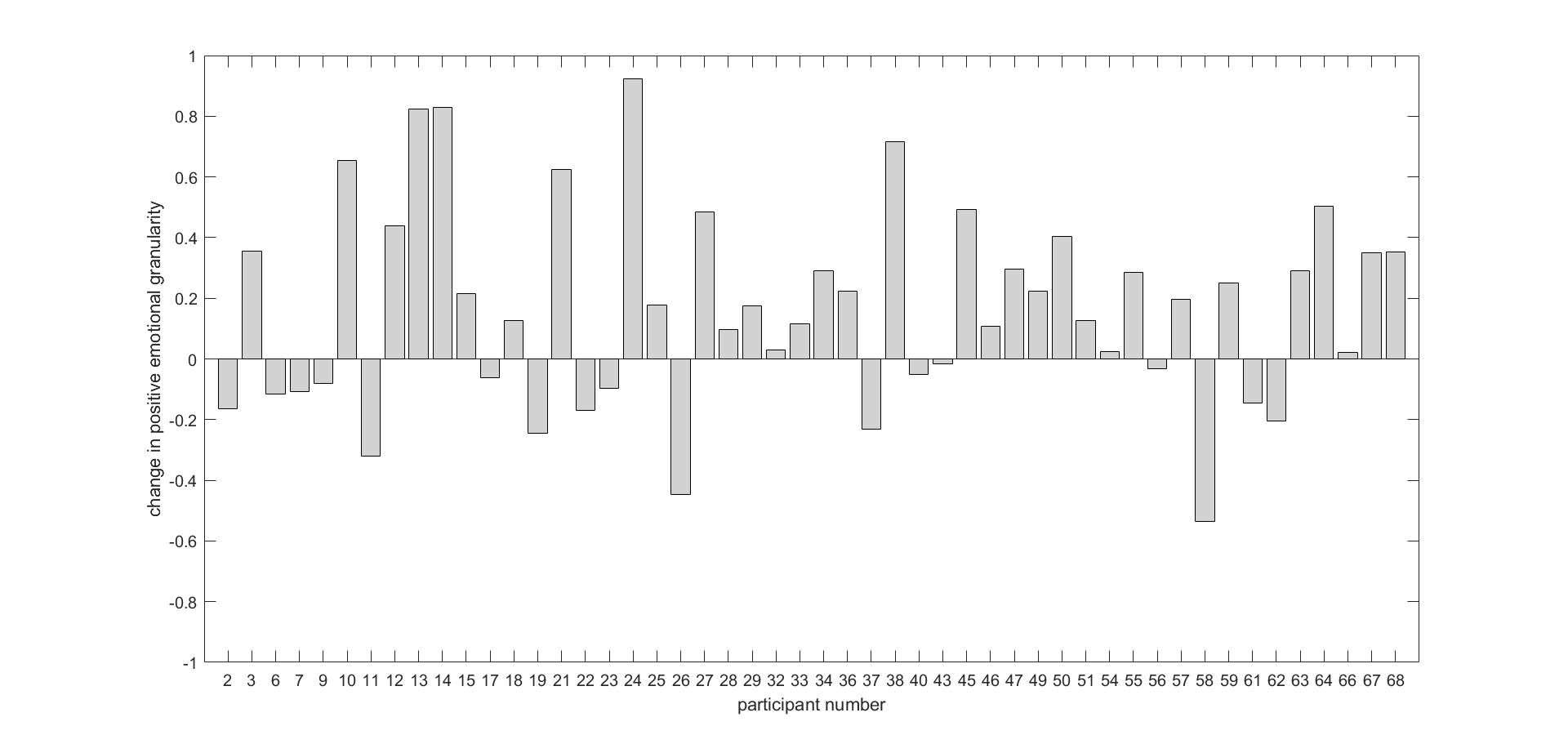


**Supplementary Figure 1.** Individual participants’ change in positive emotional granularity across assessment days, using the slope estimates from person-specific regression analyses.


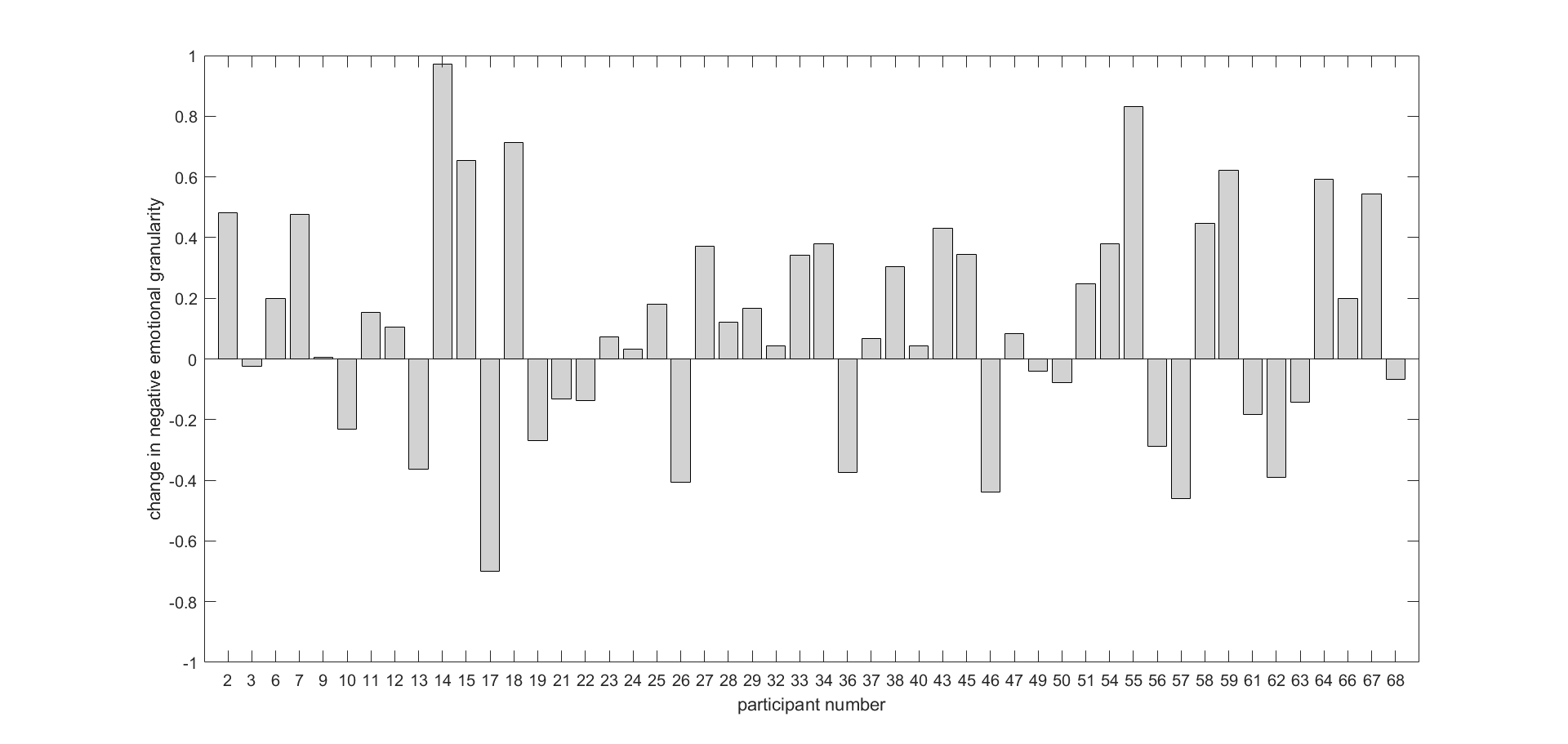


**Supplementary Figure 2.** Individual participants’ change in negative emotional granularity across assessment days, using the slope estimates from person-specific regression analyses.

**Factors Predictive of Increase in Emotional Granularity**

**Supplementary Table 2.** Bayesian Multiple Regression: Increase in Positive Granularity

| Factor | Est. β | Est. SE | 95% CI | Post. Prob. |
| --- | --- | --- | --- | --- |
| intercept | -.00 | .14 | -.27, .27 | .50 |
| nDays | .11 | .15 | -.18, .41 | .78 |
| nPrompts | .32 | .15 | .03, .62 | .98* |
| mWords | -.09 | .15 | -.37, .20 | .27 |
| mAffect | -.02 | .14 | -.30, .26 | .46 |
| mPosAffect | -.13 | .17 | -.46, .20 | .21 |
| mNegAffect | .06 | .17 | -.27, .40 | .65 |
| restingRSA | .92 | .21 | .59, 1.41 | 1.00* |

Note: * Posterior probabilities above .95 (or below .05) constitute (strong) evidence in favor of a hypothesis; ^†^ posterior probabilities above .90 (or below .10) constitute suggestive evidence in favor of a hypothesis (Franke & Roettger, 2019).

**Supplementary Table 3.** Bayesian Multiple Regression: Increase in Negative Granularity

| Factor | Est. β | Est. SE | 95% CI | Post. Prob. |
| --- | --- | --- | --- | --- |
| intercept | .00 | .13 | -.26, .26 | .50 |
| nDays | -.02 | .15 | -.31, .27 | .44 |
| nPrompts | -.53 | .15 | -.82, -.24 | .00* |
| mWords | -.20 | .14 | -.48, .08 | .07† |
| mAffect | .07 | .14 | -.20, .35 | .71 |
| mPosAffect | .11 | .16 | -.21, .43 | .75 |
| mNegAffect | -.11 | .16 | -.43, .22 | .25 |
| restingRSA | .03 | .15 | -.27, .33 | .58 |

Note: * Posterior probabilities above .95 (or below .05) constitute (strong) evidence in favor of a hypothesis; ^†^ posterior probabilities above .90 (or below .10) constitute suggestive evidence in favor of a hypothesis (Franke & Roettger, 2019).

**References**

Berntson, G. G., Quigley, K. S., Jang, J. F., & Boysen, S. T. (1990). An approach to artifact identification: Application to heart period data. *Psychophysiology*, *27*(5), 586–598. https://doi.org/10.1111/j.1469-8986.1990.tb01982.x

Carreiras, C., Alves, A. P., Lourenço, A., Canento, F., Silva, H., & Fred, A. (2018). *BioSPPy: Biosignal processing in Python*.

Franke, M., & Roettger, T. B. (2019). *Bayesian regression modeling (for factorial designs): A tutorial*. https://doi.org/10.31234/osf.io/cdxv3

Hamilton, P. (2002). Open source ECG analysis. *Computers in Cardiology*, 101–104. https://doi.org/10.1109/cic.2002.1166717

Hoemann, K., Khan, Z., Feldman, M. J., Nielson, C., Devlin, M., Dy, J., Barrett, L. F., Wormwood, J. B., & Quigley, K. S. (2020). Context-aware experience sampling reveals the scale of variation in affective experience. *Scientific Reports*, *10*, 12459. https://doi.org/10.1038/s41598-020-69180-y

Hoemann, K., Khan, Z., Kamona, N., Dy, J., Barrett, L. F., & Quigley, K. S. (2021). Investigating the relationship between emotional granularity and cardiorespiratory physiological activity in daily life. *Psychophysiology*, e13818. https://doi.org/10.1111/psyp.13818

Nabian, M., Yin, Y., Wormwood, J., Quigley, K. S., Barrett, L. F., & Ostadabbas, S. (2018). An open-source feature extraction tool for the analysis of peripheral physiological data. *IEEE Journal of Translational Engineering in Health and Medicine*, *6*, 1–11. https://doi.org/10.1109/JTEHM.2018.2878000

Qu, M., Zhang, Y., Webster, J. G., & Tompkins, W. J. (1986). Motion artifact from spot and band electrodes during impedance cardiography. *IEEE Transactions on Biomedical Engineering*, *11*, 1029–1036. https://doi.org/10.1109/tbme.1986.325869
